# Optimizing gefitinib nanoliposomes by Box-Behnken design and coating with chitosan: A sequential approach for enhanced drug delivery

**DOI:** 10.5599/admet.2366

**Published:** 2024-07-31

**Authors:** Seema Rohilla, Rajendra Awasthi, Ankur Rohilla, Sachin Kumar Singh, Dinesh Kumar Chellappan, Kamal Dua, Harish Dureja

**Affiliations:** 1 Geeta Institute of Pharmacy, Geeta University, Naultha, Panipat, Haryana, India; 2 Department of Pharmaceutical Sciences, School of Health Sciences & Technology, UPES, Dehradun, Uttarakhand, India; 3 Department of Pharmaceutical Sciences, Universal Group of Institutions, Dera Bassi, Mohali, Punjab, India; 4 School of Pharmaceutical Sciences, Lovely Professional University, Phagwara, Punjab, India; 5 Department of Life Sciences, School of Pharmacy, International Medical University, Bukit Jalil, Kuala Lumpur, Malaysia; 6 Discipline of Pharmacy, Graduate School of Health, University of Technology Sydney, NSW, Australia; 7 Faculty of Health, Australian Research Centre in Complementary and Integrative Medicine, University of Technology Sydney, Ultimo, Australia; 8 Centre for Research Impact & Outcome, Chitkara College of Pharmacy, Chitkara University, Rajpura, 140401, Punjab, India; 9 Department of Pharmaceutical Sciences, Maharshi Dayanand University, Rohtak, India

**Keywords:** Liposomes, response surface methodology, pulmonary cancer

## Abstract

**Background and Purpose:**

This study aimed to improve the stability and prolonged gefitinib release from the nanoliposomes.

**Experimental approach:**

Nanoliposomes were prepared by reverse-phase evaporation and optimized using Box-Behnken design to investigate the influence of sonication time (*X*_1_), tween 80 / soya phosphatidylcholine ratio (*X*_2_), and cholesterol/soya phosphatidylcholine ratio (*X*_3_) on nanoliposomes.

**Key results:**

Optimized nanoliposomes were quasi-spherical shaped, with a mean dimension of 93.2 nm and an encapsulation efficiency of 87.56±0.17 %. Surface decoration of the optimized batch was done using different concentrations of chitosan. The optimal chitosan concentration required to adorn the nanoliposome surface was 0.01 %. In comparison to unadorned nanoliposomes (82.16±0.65 %), adorned nanoliposomes (78.04±0.35 %) released the drug consistently over 24 h via Fickian diffusion. The IC_50_ values for surface-adorned nanoliposomes in A549 and H1299 cells were 6.53±0.75 and 4.73±0.46 μM, respectively. Cytotoxicity of the surface-decorated nanoliposomes may be due to their higher zeta potential and prolonged drug release. At the end of the sixth month, the samples stored at 4 °C were more stable than those stored at 25 °C and 45 °C. The stability of plain nanoliposomes has increased after chitosan coating. Thus, by using different concentrations of chitosan solution as coating material, we can develop a suitable sustained drug-release surface-adorned nanoliposomal formulation.

**Conclusion:**

The developed nanoliposomes may offer a new path for melanoma clinics.

## Introduction

Cancer is a group of diseases defined by the uncontrolled proliferation and metastasis of abnormal cells. It is difficult to understand the molecular causes of drug resistance, early diagnosis, multimodal psychoanalysis, and the reasons why most treatments for metastatic disease do not work [1]. Pulmonary cancer is the second most tumours, responsible for the deaths of both men and women. Imaging techniques such as magnetic resonance imaging (MRI), bone scan, computed tomography (CT), X-ray, positron emission tomography (PET), and combined PET-CT scan are used to diagnose lung cancer [2]. A combined PET-CT scan is the standard procedure among all imaging tools for assessing the size and location of lung tumors, estimating an accurate level of illness, and identifying indeterminate lung nodules [3,4]. In addition to early detection of lung cancers, appropriate therapeutic treatment is required to cure them. Treatment options for lung cancer include surgery, radiosurgery, radiotherapy, chemotherapy, and immunotherapy, depending on the stage, histological type, and patient's function. Radiation and chemotherapy can replace surgery [5]. Antagonism of the epidermal growth factor receptor (EGFR) in cancer cells disrupts normal epithelial endorsement and succession, limiting normal cell transformation into persistent and metastatic cancer cells via dysplasia to high-grade prostatic intraepithelial neoplasia (HGPIN) [6-8].

Gefitinib is a low-molecular-weight anilinoquinazoline derivative that treats pulmonary cancer [8,9]. Gefitinib binds to EGFRs to inhibit tyrosine kinase activation [10,11]. Due to its poor aqueous solubility and high permeability, it falls under Class II of the Biopharmaceutical Classification System (BCS) [12]. Low aqueous solubility limits gefitinib's oral absorption. Increasing solubility and dissolution could boost bioavailability [13-16]. To improve the solubility and oral bioavailability of poorly water-soluble drugs, different approaches were used [6,7,17]. The amphiphilic nature of liposomes (having both hydrophilic and hydrophobic parts) allows them to interact with poorly soluble drugs and water, creating a microenvironment that enhances the solubility of hydrophobic drugs. The lipid environment within the hydrocarbon chain region of the lipid bilayer of nanoliposomes can solubilize hydrophobic drugs like gefitinib, effectively increasing its aqueous solubility. Thus, nanoliposomes improve the solubility of poorly soluble drugs like gefitinib by encapsulating them in lipid bilayers, providing a suitable environment for the drug to remain dissolved and preventing aggregation and precipitation. New drug delivery systems aim to improve the curative value and safety profile of available drugs by modifying their dose frequency, bio-distribution pattern, and amount [18,19]. Due to their small size and lipoidal nature, nanoliposomes improve permeability and accumulate in tumour tissue [10,20]. Liposomes can encapsulate both water-soluble and water-insoluble drugs and release them persistently [13]. Nanoliposomes encapsulate anticancer drugs to increase systemic circulation and reduce side effects [14]. Nanoliposomes deliver the optimum drug to the targeted cells within a sufficient time to augment the time of curative action of the exposed drug [21]. This approach improved medication stability and bioavailability [22]. Regulatory agencies have recommended the use of process optimization and quality by design in the design of pharmaceutical products. These methods will help create nanoparticles with desired properties and introduce a reproducible, robust scale-up method. Nanoliposomes protect the active therapeutic molecule against chemical degradation and increase the rate of component release [23]. Encapsulating gefitinib in liposomes addresses critical issues related to its poor solubility, bioavailability, and stability. This approach enhances the therapeutic efficacy of gefitinib, reduces side effects, enables targeted and controlled drug delivery, and potentially overcomes drug resistance, making it a valuable strategy in cancer treatment.

The formulation protocol is optimized by using different designs such as Box-Behnken Design (BBD), and D-optimal design of response surface methodology (RSM). In the present study, the BBD was used to optimize the process, as it produced fewer runs than the central composite design with three variables [24-26]. The BBD has many advantages over other experimental designs. In this, a minimum of three levels of independent variables must be required to run, and the divergence from the predictable outcomes at any point is identified exclusively by its expanse from the design centre point [27]. Thus, it reduces the cost associated with the expensive critical methods by reducing their computational noise. The BBD used the response surface methodology to select the optimal responses for a precise reaction [28].

Chitosan is an N-deacetylated derivative of chitin and contains chiefly glucosamine units. It acts like a polycationic polymer due to the presence of amino groups. It has anti-inflammatory and antioxidant properties [29,30]. The destructive effects of gastrointestinal tract pH, bile salts, and pancreatic lipase offered a great challenge for liposomal delivery systems. Chitosan or related polymers are used as coating materials to improve the stability of nano-formulations and achieve targeted payload delivery [31]. Literature demonstrated that an amendment in the ratio of the lipid-based and polymer-based systems led to the development of a new system [32]. Positively charged chitosan forms an ionic bond with the negatively charged nanoliposomes to produce a uniform coating on the nanoliposome surface [33,34]. This coating rendered them mucoadhesive and augmented their stability profile [30,34]. Coating with polymers of desired characteristics is a confirmed method to modify the surface properties of nanocarriers. To coat, nanoliposomes are simply mixed with a polymer solution. This improves the mutual repulsion among the adjacent bilayers during storage and thus improves the stability of nanoliposomes. Different synthetic and natural polymers have been used to modify the exterior character of nanoliposomes. We find chitosan to be the most suitable in this context because of its positive charge, and we assume it has an optimistic future in the pharmaceutical field.

In the present study, gefitinib-loaded nanoliposomes were prepared by the reverse phase evaporation method and optimized using BBD. The potential rationale for conducting this study was to scrutinize the consequences of sonication time (*X*_1_), the ratio of tween 80 to soya phosphatidylcholine (*X*_2_), and the ratio of cholesterol to soya phosphatidylcholine (*X*_3_) on particle size and entrapment efficiency of gefitinib-loaded nanoliposomes. The optimum batches were then encrusted by using varying concentrations of chitosan solutions. The optimum concentration for the coating of nanoliposomes was found to be 0.01 % as reported in our previous communication [30]. Different techniques like FTIR studies, DSC analysis, zeta potential studies, transmission electron microscopy, and particle size analysis were used to confirm the presence of chitosan coating on nanoliposomes. Further optimization of nanoliposomes was done for their systemic therapeutic purposes by characterizing the uncoated and surface-adorned nanoliposomes. The MTT assay was used to test the cytotoxicity of gefitinib-loaded nanoliposomes and chitosan-coated gefitinib nanoliposomes on a human non-small cell lung carcinoma cell line (H1299) and human adenocarcinomic alveolar basal epithelial cells (A549). ICH guidelines were followed to perform the stability studies on optimized batches.

## Experimental

### Materials

Mac Chem Products India Pvt. Ltd., Mumbai, India, gifted gefitinib sample. Dimethyl sulfoxide, Tween 80, potassium dihydrogen orthophosphate, and diethyl ether were obtained from Loba Chemie, India. Cholesterol was purchased from Sigma Aldrich, MO, USA. Soya phosphatidylcholine was purchased from Central Drug House (P) Ltd., New Delhi, India. Chitosan was purchased from Fluka Chemie, Germany. Ammonium acetate and acetonitrile (HPLC-grade) were procured from Rankem, India, and Fisher Scientific, Mumbai, respectively. Human lung carcinoma cells (A549 and H1299) were provided by the National Cell Repository, Pune, India. Each ingredient used in the research was of analytical grade.

### Preparation of gefitinib-loaded nanoliposomes and coating

Gefitinib-loaded nanoliposomes were formulated by the reverse-phase evaporation technique using BBD. Soya phosphatidylcholine and cholesterol were liquefied in diethyl ether. Gefitinib was dissolved in distilled water using ethanol as a cosolvent. The aqueous and organic parts were mixed, followed by ultrasonication with a probe sonicator (Sonics Vibra Cell, USA, VCX750W) on an ice bath for 5 min with 1 s on and 1 s off intermission. The round-bottom flask in the rotary evaporator was used to transfer the consequential emulsion and to convert it into gel under reduced pressure at 40 °C. Ten millilitres of phosphate buffer (0.10 M, pH 7.0) containing Tween 80 was poured into a round-bottom flask with moderate vortexing to break the gel into liposomes under the atmosphere of nitrogen gas at room temperature to relinquish the resting fumes of diethyl ether. The liposomes were then reduced to nanosize by ultrasonication on an ice bath with a probe sonicator for 10 minutes with 1 s on and 1 s off intermission. The product was centrifuged at 3500 rpm for 20 min, following an incubation period of 12 h at room temperature [35,36]. The sterile, double-distilled, deionized water is used to wash the pellets and then centrifuged. After subsequent washing and centrifugation, the resulting product was re-suspended in sterilized, double-distilled, deionized water. The best-selected nanoliposomes were surface adorned (coated) by magnetically stirring a suitable amount of chitosan solution (0.01 % w/v) into the nanoliposomal suspension. After 1 hour of stirring, the product was incubated overnight at 4 °C [37,38].

### Box-Behnken design

The BBD was used to study the effect of three independent variables: *X*_1_ - sonication time, min, *X*_2_ - tween 80 / soya lecithin weight ratio, w/w, and *X*_3_ – cholesterol/soya lecithin weight ratio, on two dependent variables: *Y*_1_ - particle size and *Y*_2_ - encapsulation efficiency.

### Mathematical model

BBD is a specially designed model for the response surface methodology. Every factor requires only three levels to fit in a second-order regression (quadratic) model. The BBD sets an intermediate level of factors, avoiding the extreme axial (star) points as in the central composite design (CCD). Furthermore, the BBD uses facade points that are more realistic than the corner points in the CCD. The number of experimental runs is small. For analysis, the BBD required three factors at three levels. It is critical to identify appropriate estimation methods to compute the useful relationship between the response surface and independent variables. Generally, a second-order polynomial equation is used in response surface methodology and is expressed as Equation 1 [26-28]:



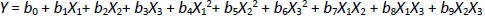

(1)


where *Y* is the level of the response variable. *b*_0_ is the value of the desired response at the center point of the design. *b*_1_, *b*_2_, and *b*_3_, are regression coefficients of linear terms. *b*_4_, *b*_5_, and *b*_6_ are regression coefficients of quadratic terms. *b*_7_, *b*_8_, and *b*_9_ are the regression coefficients of interaction terms. *X*_1_, *X*_2_, and *X*_3_ are the dimensionless coded variables. The second-order model includes all terms of the first-order model, along with all quadratic terms like b_4_*X*_1_^2^ and all cross-product terms like *b*_8_*X*_1_*X*_3_.

### Characterization of nanoliposomes

#### Encapsulation efficiency and loading efficiency

To determine the percentage of encapsulated drug, nanoliposomes were mixed with alcohol and sonicated. After dilution with the mobile phase in 1:1 fraction, the samples were centrifuged at 10,000 rpm for 5 minutes. The quantity of drug was analysed using high-performance liquid chromatography (HPLC) (Agilent Technologies 1200 Series, Germany; quaternary pump; Eclipse XDB-C18 column (4.6×150 mm) packed with octadecylsilane and porous silica (3 μm) at 254 nm. The mobile phase consisting of 1 % ammonium acetate and acetonitrile at 2:3 ratio was analyzed at a 1 mL/min flow rate. The temperature was maintained at 25 °C [39,40]. The test sample was introduced via a microsyringe and examined at 254 nm. Equations 2 and 3 were used to calculate the percentage encapsulation efficiency and percentage loading, respectively:



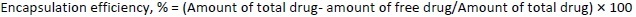

(2)




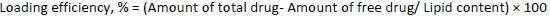

(3)


#### Determination of particle size, polydispersity index and zeta potential

Zeta potential, mean particle size, and size distribution were analysed by quasielastic laser light scattering using a Malvern Zetasizer® (Zeta Sizer, Nano ZS90, Malvern Instruments, UK). The sample was scanned at 64 runs in a polystyrene cuvette of the hydro-dispersing unit of Zetasizer to determine the particle dimension and polydispersity index. The particle size distribution was estimated by the polydispersity index (PDI) value [41,42].

#### Differential scanning calorimetry

A differential scanning calorimeter (DSC) (Q 10, TA Instruments, New Castle, United States) was used for thermal analysis to determine the melting point, purity, and encapsulation of the drug in nanoliposomes before and after coating. The apparatus was standardized with indium. The aluminium pans were used to load the trial formulation. The temperature was altered progressively from 25 to 300°C at a rate of 10 °C/min in an atmosphere of nitrogen (60 mL/ min). An empty pan was used as a reference under the same conditions [42].

#### Fourier transforms infrared spectroscopy

Fourier transforms infrared (FT-IR) spectra of pure gefitinib, gefitinib-loaded unadorned nanoliposomes, and surface-adorned nanoliposomes were recorded using an FT-IR-Alpha Bruker 1206 0280. The sample holder was cleaned, and background measurements were done to nullify the effect of environmental impurities. The samples were positioned on a holder for analysis and scanned from 400 to 4000 cm^-1^ at a resolution of 4 cm^-1^ [43].

#### Morphological characterization

Morphological characterization of the sample suspension was done using a transmission electron microscope (H-600 TEM, Hitachi Ltd., Tokyo, Japan) at 200 kV. A drop of the diluted sample was placed on a carbon-coated copper grid and dried properly [44].

#### Determination of gefitinib release and release kinetics from nanoliposomes

Drug release studies were carried out using a dialysis bag method in simulated intestinal fluid (pH 6.8), acetate buffer (pH 4.0), and 0.1 N HCl (pH 1.2) [45]. Gefitinib-loaded nanoliposomes and surface-adorned nanoliposomes (each containing 250 mg of the drug) were sealed in the preactivated dialysis bag (molecular weight cutoff, 12,000 Da, Himedia, Mumbai, India). A sample containing a dialysis bag was immersed in the USP dissolution apparatus II (DS 8000, Lab India, Mumbai, India) containing 200 mL of dissolution medium. The temperature was kept constant at 37±0.5 °C and the paddle was rotated at 100 rpm. At predetermined intervals (0.5, 1, 2, 4, 6, 8, 12, 18 and 24 h), 2 mL of the samples were collected. The fresh preheated medium was used to maintain the sink condition. Following filtration via a 0.45 μm filter, the samples were analysed using HPLC (1200 series, Agilent Technologies, Germany) at 254 nm. The sample analysis was done in triplicate, and mean values were used for estimation [46]. *In vitro* drug release data was fitted into zero-order, first-order, Higuchi's, and Korsmeyer-Peppas’ models to determine drug release kinetics and mechanism from the nanovesicles [47].

### Cytotoxicity study

#### Cell culture and treatment

Dulbecco's Modified Eagle's Medium with 2 % antibiotics and 10 % fetal bovine serum was used to culture A549 and H1299 human lung cancer cells. The cells were grown at 37 °C in 5 % CO_2_ and 95 % humidity for 24 h. Cells were spread uniformly (8,000 to 10,000 per 96-well plate). After washing with phosphate-buffered saline, cells were cultured in a serum-free medium for 2 days. Different concentrations (1, 5, and 20 μM) of the samples were mixed with dimethyl sulfoxide and tested against cancer cells. Pure drug was used as a control. The experiment was conducted in triplicate [48].

#### MTT assay

The MTT (3-(4, 5-dimethylthiazol-2-yl)-2, 5-diphenyl tetrazolium bromide) assay was carried out using a 96-well plate technique [40,41]. A549 and H1299 cells were counted using an automated cell counter. The cells were exposed to synthetic chemicals for two days after being seeded with approximately 8,000 to 10,000 cells in each well of the 96-well plates and 100 μL of media in each space. After two days, the media was removed and washed with phosphate-buffered saline. The cells were treated with doses of 1 μM, 5 μM, and 25 μM and after 1 day, and they were incubated for 48 h. Media was replaced with 10 μL of MTT solution (5 mg/ 10 mL of phosphate-buffered saline) and then left to incubate for 4 hours in the dark. The MTT solution was then removed from each well, 100 μL of dimethyl sulfoxide solution was added to the intracellular precipitates, and the absorbance of the violet hue created by the addition of dimethyl sulfoxide was measured spectrophotometrically at 570 nm. Half of the inhibitory concentration (*IC*_50_), at which 50 % of the cells were confirmed to be viable, was used to represent the study results. The proportion of viable cells was calculated using Equation 4 [49].



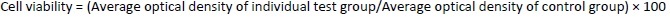

(4)


#### Stability studies of nanoliposomal system

The samples were stored at different temperatures according to ICH guidelines for six months to determine the stability of the unadorned and surface-adorned nanoliposomal preparations, including freeze temperature (4±1 °C), room temperature (25±2 °C / 60±5 % of relative humidity (RH), and higher temperature (45±2 °C/ 75±5 % RH). The storage stability was assessed by monitoring changes in the percentage of encapsulation, particle size, and physical appearance [36,39].

## Results

### Loading efficiency, entrapment efficiency, and drug content

**Figure 1.**
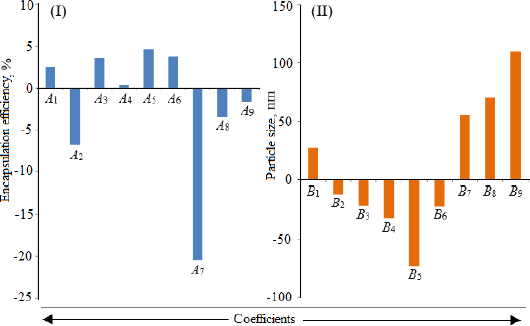
(I) Upshot plot of coefficients on encapsulation efficiency [*A*_1_, *A*_2_, and *A*_3_ are coefficients of main effects (*X*_1_, *X*_2_, and *X*_3_); *A*_4_, *A*_5_, and *A*_6_ are coefficients of interaction terms (*X*_1_*X*_2_, *X*_2_*X*_3_, and *X*_1_*X*_3_); and *A*_7_, *A*_8_, and *A*_9_ are coefficients of square terms (*X*_1_^2^, *X*_2_^2^, *X*_3_^2^)]. (II) Upshot plot of particle size [(*B*_1_, *B*_2_, and *B*_3_ are coefficients of main effects (*X*_1_, *X*_2_, and *X*_3_); *B*_4_, *B*_5_, and *B*_6_ are coefficients of interaction terms (*X*_1_*X*_2_, *X*_2_*X*_3_, and *X*_1_*X*_3_); and *B*_7_, *B*_8_, and *B*_9_ are coefficients of square terms (*X*_1_^2^, *X*_2_^2^, *X*_3_^2^)].

**Figure 2.**
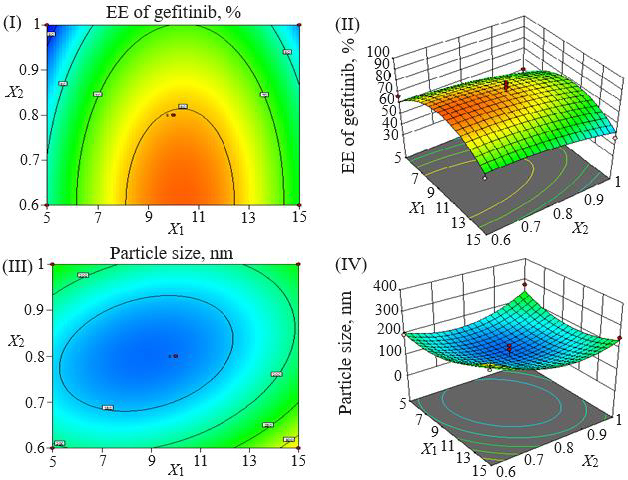
Response surface plots and contour plots showing the influence of level of sonication time, Tween 80/soya lecithin ratio, and cholesterol/soya lecithin ratio on mean particle size (I, II) and encapsulation efficiency (III, IV).

Loading efficiency, encapsulation efficiency, and drug content ranged from 24.50±0.33 to 34.91±0.32 %, 47.00±0.13 to 87.56±0.17 %, and 50.23±0.38 to 89.76±0.51 %, respectively (Table 1). Formulation NG13 had the maximum loading and encapsulation efficiencies (34.91±0.32 and 87.56±0.17 %, respectively). Figures 1 and 2 display the Pareto chart, 3D-response surface plots, and corresponding contour plots illustrating the efficiency of nanoliposome encapsulation. Quadratic equations were developed to represent the relationship between the dependent and independent variables (Equation 5 and 6).

**Table 1.** Levels of independent parameters used in Box-Behnken design and results of *in vitro* characterization of gefitinib-loaded nanoliposomes.

Formulation code	*X*_1_ (min)*	*X*_2_ *	*X*_3_ *	Encapsulation efficiency, %	Loading efficiency, %	Particle size,nm	PDI	Drug content, %	Zeta potential, mV
NG1	-1(5)	-1 (0.6)	0 (0.2)	66.70±0.14	31.09±0.21	195.00	0.288	70.97±0.73	-8.37
NG2	+1(15)	-1 (0.6)	0 (0.2)	61.20±0.07	29.81±0.22	294.80	0.297	64.29±0.42	-7.98
NG3	-1(5)	+1 (1.0)	0 (0.2)	51.15±0.12	25.58±0.32	275.60	0.287	54.76±0.62	-7.43
NG4	+1(15)	+1 (1.0)	0 (0.2)	47.00±0.13	24.50±0.33	247.80	0.256	50.23±0.38	-9.75
NG5	-1(5)	0 (0.8)	-1 (0.1)	50.48±0.13	24.69±0.24	160.48	0.376	53.76±0.44	-9.12
NG6	+1(15)	0 (0.8)	-1 (0.1)	56.20±0.15	28.95±0.21	380.48	0.425	59.96±0.51	-7.72
NG7	-1(5)	0 (0.8)	+1 (0.3)	51.37±0.17	26.67±0.16	351.37	0.427	54.28±0.78	-7.34
NG8	+1(15)	0 (0.8)	+1 (0.3)	75.46±0.11	32.23±0.17	278.96	0.428	78.54±0.87	-6.97
NG9	0(10)	+1 (1.0)	+1 (0.3)	83.14±0.13	34.36±0.15	383.14	0.422	85.67±0.95	-6.78
NG10	0(10)	+1 (1.0)	-1 (0.1)	63.25±0.10	30.90±0.14	363.25	0.422	67.49±0.54	-7.65
NG11	0(10)	-1 (0.6)	+1 (0.3)	79.85±0.12	33.13±0.18	296.50	0.410	81.76±0.78	-7.32
NG12	0(10)	-1 (0.6)	-1 (0.1)	74.96±0.15	31.73±0.32	186.70	0.242	77.43±0.42	-7.02
NG13	0(10)	0 (0.8)	0 (0.2)	87.56±0.17	35.16±0.31	93.20	0.233	89.76±0.51	-6.46
NG14	0(10)	0 (0.8)	0 (0.2)	85.96±0.15	34.89±0.33	179.60	0.236	88.56±0.63	-6.76
NG15	0(10)	0 (0.8)	0 (0.2)	74.46±0.14	33.62±0.31	160.90	0.245	87.32±0.51	-6.67
NG16	0(10)	0 (0.8)	0 (0.2)	70.97±0.18	31.89±0.34	101.60	0.235	79.56±0.64	-6.47
NG17	0(10)	0 (0.8)	0 (0.2)	83.18±0.16	34.91±0.32	101.90	0.237	81.57±0.72	-6.48

*Actual values are given in brackets



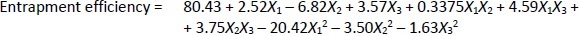

(5)


where *X*_1_, *X*_2_, and *X*_3_ are the main effects; *X*_1_*X*_2_, *X*_2_*X*_3_, and *X*_1_*X*_3_ are the interaction terms; and *X*_1_^2^, *X*_2_^2^, *X*_3_^2^ are square terms. The percentage encapsulation efficiency's F-value (4.62) and p-value (0.0281) indicated that the model is significant.

### Particle size, polydispersity index and zeta potential

The particle size ranged between 93.2 and 490.0 nm, whereas the polydispersity index values varied from 0.233 to 0.428 for all formulations. Batch NG13 had a minimum particle size of 93.2 nm with a PDI of 0.233 (Table 1). The zeta potential of the optimized batch was found to be - 6.51±0.03 mV. However, after coating, the value of zeta potential was found to be 23.7±0.03 mV. A Pareto chart was prepared to analyse the effect of coefficients on particle size (Figure 1). The 3D response surface plots are depicted in Figure 2. The polynomial equation was generated using Design Expert® Software. The particle size F-value (3.95) and p-value (0.0418) indicated that the model is significant. An excellent agreement was found between the actual value and the predicted value, which indicates the validity of the developed model (Table 2).

**Table 2.** Actual and predicted values for all response variables for different batches.

Formulation code	Mean particle size, nm		Encapsulation efficiency, %		Cumulative drug release, %	
	Actual	Predicted	Actual	Predicted	Actual	Predicted
NG1	179.60	127.44	85.96	80.43	81.96	79.72
NG2	195.00	217.20	66.70	61.15	68.88	68.83
NG3	101.60	127.44	70.97	80.43	75.69	79.72
NG4	93.20	127.44	87.56	80.43	82.16	79.72
NG5	351.37	318.29	51.37	54.84	76.90	76.82
NG6	101.90	127.44	83.18	80.43	81.43	79.72
NG7	363.25	352.37	63.25	61.16	79.80	79.67
NG8	160.90	127.44	74.46	80.43	77.34	79.72
NG9	247.80	225.60	47.00	52.55	73.92	73.97
NG10	278.96	246.38	75.46	69.06	76.90	76.69
NG11	186.70	241.48	74.96	75.81	81.57	81.73
NG12	287.90	266.20	51.15	46.84	71.63	71.56
NG13	294.80	316.50	61.20	65.51	69.59	69.67
NG14	250.40	282.98	50.48	56.88	73.39	73.60
NG15	383.14	328.36	83.14	82.30	76.90	76.74
NG16	296.50	307.38	79.85	81.94	77.48	77.61
NG17	380.48	413.56	56.20	52.73	76.90	76.98



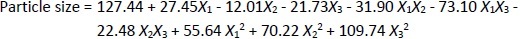

(6)


The *p*-values less than 0.0500 indicate that the model terms are significant. Values greater than 0.1000 indicate the model terms are insignificant (Table 3). The ANOVA data of the quadratic model for encapsulation efficiency (%) implies the model is significant with a model *F*-value of 4.62. In this case, *X*_2_ and *X*_1_² are significant model terms. The lack of fit F-value of 1.34 implies the lack of fit is insignificant relative to the pure error. There is a 38.00 % chance that a large lack of fit *F*-value could occur due to noise. The *R*², adjusted *R*², and predicted *R*² values for the ANOVA data of encapsulation efficiency were 0.8558, 0.6704 and -0.2682 %, respectively. Adequate precision (signal-to-noise ratio) greater than 4 is desirable. A ratio of 5.905 indicates an adequate signal. In the case of particle size, the model F-value of 3.95 implies the model is significant. In this case, *X*_1_*X*_3_, *X*_2_², and *X*_3_² are significant model terms. The lack of fit F-value of 4.12 implies that it is not significant relative to the pure error. There is a 10.24 % chance that a large lack of fit F-value could occur due to noise. The *R*², adjusted R², and predicted R² values for the ANOVA data of encapsulation efficiency were 0.8356, 0.6243, and -1.0500 %, respectively. The ratio of adequate precision (6.1640) indicates an adequate signal.

**Table 3.** ANOVA results for quadratic model of particle size, encapsulation efficiency and cumulative drug release.

Source	Sum of squares	*df*	Mean square	*F*-value	*p*-value	Remark
Encapsulation efficiency, %						
Model	2546.21	9	282.91	4.62	0.0281	Significant
*X*_1_	50.80	1	50.80	0.8289	0.3929	
*X*_2_	371.69	1	371.69	6.06	0.0433	
*X*_3_	102.03	1	102.03	1.66	0.2380	
*X*_1_ *X*_2_	0.4556	1	0.4556	0.0074	0.9337	
*X*_1_ *X*_3_	84.36	1	84.36	1.38	0.2791	
*X*_2_ *X*_3_	56.25	1	56.25	0.9178	0.3700	
*X*_1_²	1755.35	1	1755.35	28.64	0.0011	
*X*_2_²	51.45	1	51.45	0.8394	0.3900	
*X*_3_²	11.19	1	11.19	0.1826	0.6820	
Residual	429.04	7	61.29			
Lack of fit	214.92	3	71.64	1.34	0.3800	Not significant
Pure error	214.11	4	53.53			
Cor total	2975.25	16				
Particle size, nm						
Model	131800.00	9	14640.44	3.95	0.0418	Significant
*X*_1_	6027.47	1	6027.47	1.63	0.2427	
*X*_2_	1154.16	1	1154.16	0.3117	0.5940	
*X*_3_	3776.67	1	3776.67	1.02	0.3461	
*X*_1_ *X*_2_	4070.44	1	4070.44	1.10	0.3292	
*X*_1_ *X*_3_	21375.90	1	21375.90	5.77	0.0473	
*X*_2_ *X*_3_	2020.95	1	2020.95	0.5458	0.4841	
*X*_1_²	13036.16	1	13036.16	3.52	0.1027	
*X*_2_²	20759.99	1	20759.99	5.61	0.0498	
*X*_3_²	50706.81	1	50706.81	13.70	0.0076	
Residual	25917.12	7	3702.45			
Lack of fit	19584.50	3	6528.17	4.12	0.1024	Not significant
Pure error	6332.61	4	1583.15			
Cor total	157700.00	16				
Cumulative drug release, %						
Model	235.34	9	26.15	5.08	0.0217	Significant
*X*_1_	5.30	1	5.30	1.03	0.3439	
*X*_2_	24.75	1	24.75	4.81	0.0643	
*X*_3_	4.29	1	4.29	0.8346	0.3913	
*X*_1_ *X*_2_	0.6241	1	0.6241	0.1213	0.7378	
*X*_1_ *X*_3_	3.08	1	3.08	0.5989	0.4643	
*X*_2_ *X*_3_	0.3540	1	0.3540	0.0688	0.8006	
*X*_1_²	142.28	1	142.28	27.66	0.0012	
*X*_2_²	35.36	1	35.36	6.88	0.0343	
*X*_3_²	18.91	1	18.91	3.68	0.0967	
Residual	36.00	7	5.14			
Lack of fit	0.2017	3	0.0672	0.0075	0.9990	Not significant
Pure error	35.80	4	8.95			
Cor total	271.35	16				

The model *F*-value of 5.08 implies that the developed model for cumulative drug release is significant. In this case, *X*_1_² and *X*_2_² are significant model terms. The lack of fit *F*-value of 0.01 implies the lack of fit is not significant relative to the pure error. There is a 99.90 % chance that a large lack of fit *F*-value could occur due to noise. The *R*², adjusted *R*², and predicted *R*² values for the ANOVA data of encapsulation efficiency (%) were 0.8673, 0.6967, and 0.7820, respectively. The predicted *R*² of 0.7820 is in reasonable agreement with the adjusted *R*² of 0.6967; *i.e.*, the difference is less than 0.2. The ratio of 7.4158 indicates an adequate signal. The results suggested that the developed models can be used to navigate the design space.

### Thermal analysis

**Figure 3.**
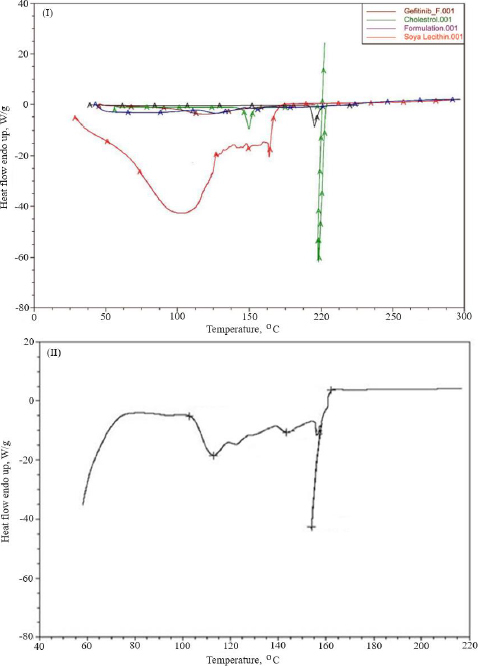
Overlay of DSC analysis of gefitinib, chitosan, cholesterol, soya lecithin, and uncoated nanoliposomal formulation (I), and chitosan-coated optimized nanoliposomes (formulation NG13) (II).

Thermal analysis was performed to verify the amorphous or crystalline performance of gefitinib (before and after entrapment in nanoliposomes). In the DSC thermogram, a spiky endothermic peak was shown at 197.51 °C, within the gefitinib melting range of 193-198 °C, with a purity level of 99.42 mol % (Figure 3I). The endothermic peak of the drug disappeared in the thermogram of batch NG13 (Figure 3II). At 153.87°C, a sharp endothermic peak almost exactly matches the liquefaction point of cholesterol. A prominent endothermic peak appeared in the case of surface-adorned nanoliposomes. The disappearance of the endothermic peak of the drug in the case of an optimized batch signifies that during the preparation of nanoliposomes, the drug may disperse as an amorphous form in the polymeric medium.

### Fourier transforms infrared spectrophotometry

**Figure 4.**
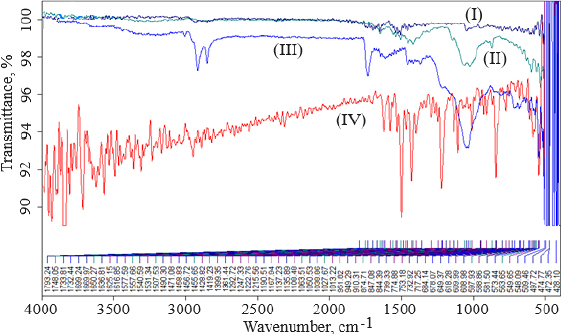
Overlay of FTIR spectra of cholesterol (I), chitosan (II), soya lecithin (III), and (IV) gefitinib.

**Figure 5.**
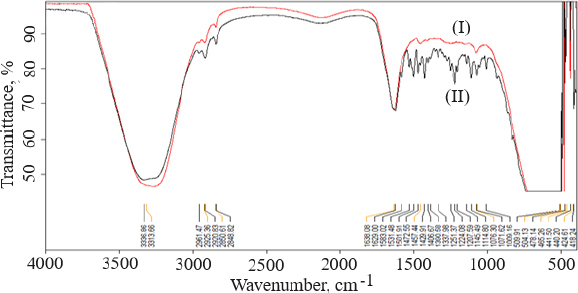
Overlay of FTIR spectra of unadorned nanoliposomal formulation (I); coated nanoliposomal formulation (II).

FTIR data can provide comprehensive information about the chemical identity, structure, purity, and interactions of the sample being analyzed. Figure 4 shows FTIR spectra of cholesterol (I), chitosan (II), soya lecithin (III), and (IV) gefitinib. The overlay spectra of gefitinib-loaded nanoliposomes and surface-adorned nanoliposomes are shown in Figure 5. The FT-IR spectrum of gefitinib illustrated the characteristic peaks at 1398.46 cm^-1^ (C=N stretching), 1470.88 cm^-1^ (C-H deformation), 1623.93 cm^-1^ (C=O stretching), 2852 cm^-1^ (=CH_2_ stretching), 2941.83 cm^-1^ (N-CH_3_ stretching), and 3397.08 cm^-1^ (N-H stretching). The strong and wide peak in the range of 3500-3300 cm^-1^ corresponds to N-H stretching and hydrogen-bonded stretching vibrations from primary amine. The -CH stretching was depicted by the peak in the range of 2923.28-2850.91 cm^-1^. In the chitosan spectra (Fig. 4II), the broad peak in the range of 1637.39-1419.23 cm^-1^ corresponded to carbonyl amide (amide I). A broad peak in the 1063.51-1027.67 cm^-1^ range almost corresponds to the COC groups symmetric stretching of the chitosan molecule, which is depicted in the FT-IR spectra of chitosan-coated nanoliposomes (Fig. 5II) but not in the spectrum of unadorned nanoliposomes (I). Figure 5 demonstrated that neither new peaks nor considerable alteration of functional peaks nor overlaps of attribute peaks were observed in the optimized batch and surface-adorned nanoliposomes compared to the pure drug spectrum.

#### Morphology

**Figure 6.**
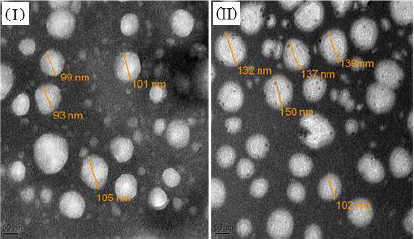
Transmission electron micrographs of unadorned nanoliposomes (I), surface adorned nanoliposomes (II).

Transmission electron micrographs of unadorned nanoliposomes and surface-adorned nanoliposomes are presented in Figure 6. The results for batch NG13 showed that the particles are ellipsoidal or quasi-spherical unilamellar nanoliposomes. The coating of chitosan was clearly visible on the surface of surface-adorned nanoliposomes in TEM images. TEM images did not show any physical aggregation of particles.

### Cumulative drug release and release kinetics

**Figure 7.**
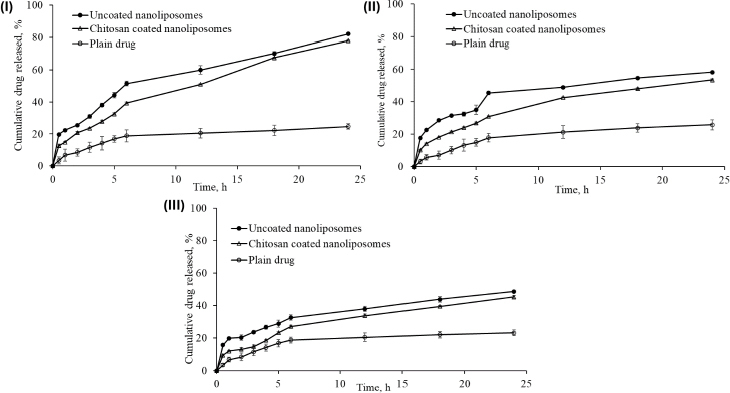
Comparison of *in vitro* release of drug from pure drug dispersion, optimized uncoated nanoliposomes and chitosan-coated nanoliposomes (I) in simulated gastrointestinal fluid, (II) in acetate buffer pH 4.0, and (III) in simulated intestinal fluid pH 6.8. Data presents mean ± SD, *n* = 3.

After 24 h of dissolution study, the percentage cumulative drug release in simulated gastrointestinal fluid (pH 1.2), acetate buffer (pH 4.0), and simulated intestinal fluid (pH 6.8) was 82.16±0.65, 58.25±0.39 and 48.72±0.55 %; and 78.04±0.3499, 53.54±0.5916 and 45.31±0.9376 %, respectively, from uncoated nanoliposomes and chitosan-coated nanoliposomes. This may be attributed to the small particle size of prepared nanoliposomes (93.2 nm), which provides a larger effective surface area for drug release and a short diffusion distance for the rapid release of the entrapped drug. pH influenced the release behaviour of nanoliposomal formulations. The pure drug dispersion showed a maximum drug release of 25.82±29 % in acetate buffer (pH 4.0) after 24 h. The comparison of cumulative drug release between pure drug solution, optimised uncoated and surface-adorned nanoliposomal formulations is shown in Figure 7.

Slow drug release was recorded for up to 5 hours in the dissolution study. After hydrating the polymer, nanoliposomes released the drug following a first-order drug release pattern. The drug was released more consistently from the surface-adorned formulations than optimised uncoated nanoliposomal formulations.

The values of correlation coefficient obtained for zero order, first order, Higuchi’s release model, and Korsemeyer Peppas’s model were found to be 0.924, 0.981, 0.980, and 0.968, respectively, in uncoated nanoliposomes, whereas in surface-adorned nanoliposomes, the values of correlation coefficient were found to be 0.974, 0.993, 0.978, and 0.953, respectively, for zero order, first order, Higuchi’s release model, and Korsemeyer Peppas’s model. The elevated correlation coefficients in first-order release kinetics for both uncoated and surface-adorned nanoliposomes, such as 0.981 and 0.993; 0.905 and 0.965; and 0.958 and 0.959 in simulated gastrointestinal fluid (acetate buffer pH 4.0) and simulated intestinal pH 6.8, demonstrated that drug release followed first-order release kinetics. For Korsmeyer-Peppas’ model, the n values were found to be 0.395, 0.327 and 0.292 in simulated gastrointestinal fluid, in acetate buffer (pH 4.0), and simulated intestinal fluid (pH 6.8), respectively, for uncoated nanoliposomes. The n values were 0.435, 0.419, and 0.426 in simulated gastrointestinal fluid, acetate buffer pH 4.0, and simulated intestinal fluid pH 6.8 for surface-adorned nanoliposomes, respectively.

### Cytotoxicity studies

**Figure 8.**
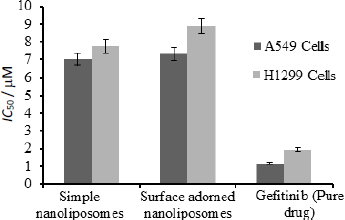
*IC*_50_ value of pure gefitinib, simple nanoliposomes (uncoated), and surface-adorned nanoliposomes.

Figure 8 depicts the half-inhibitory concentrations of free gefitinib and uncoated and surface-adorned nanoliposomes in H1299 and A549 cells. After incubation, the values of half inhibitory concentration for free gefitinib were 1.17±0.8 and 1.96±0.76 μM, respectively, in A549 and H1299 cells. In contrast, for uncoated and surface adorned nanoliposomes in A549 and H1299 cells, the values were found to be 7.03±0.85, 7.74±0.75 and 7.31±0.98, 8.89±0.97 μM, respectively. Chitosan-coated nanoliposomes, on the other hand, had a slightly higher inhibitory potential than uncoated formulations. The values of half inhibitory concentration for chitosan-coated nanoliposomes in A549 and H1299 cells were found to be 7.74±0.75, 8.89±0.97 μM, respectively. In the current study, the results showed that coated formulations (23.7±0.03 mV) had a higher zeta potential than unadorned formulations (-6.51±0.03 mV).

### Stability studies

The results of the stability study demonstrated that 4 °C was the optimum temperature to retain the chemical and physical stability of formulated vesicles for 6 months. While conducting the stability study, the samples kept at 25 and 45 °C showed many alterations in encapsulation efficiency, particle size, and physical appearance, but the samples kept at 4 °C remained stable. A noteworthy decline in the entrapment efficacy was estimated, which depicts a considerable escape of the drug from the preparations over time. The mean particle size of preparations was also estimated as a function of time and temperature. Entrapment efficacy of the nanovesicles kept at 4°C decreased by 9, 19, 32, and 40 %, whereas those kept at 25 °C decreased by 16, 34, 60, and 87 %, while those kept at 45 °C decreased by 43, 66, 81, and 94 % at the end of 1, 2, 3, and 6 months, respectively.

The physical appearance of nanoliposomal preparations was observed at 4, 25 and 45 °C for the first, second, third, and sixth months. Following the first month, the sample at 45 °C showed an alteration in the colour and viscosity of the preparations with a small accretion of particles, but they were dispersed on quaking. However, the samples kept at 4 and 25 °C were found to be acceptable. Traces of fungal growth were observed above the formulation horde at 45 °C in the latter part of the second month, prompting the decision to end the batch's studies after two months. Nanoliposomes stored at 4 and 25 °C showed no discernible changes in particle dimension when compared to nanoliposomes stored at 45 °C. The sample stored at 25 °C had traces of fungal growth as of the end of the third month, while batches at 4 °C were found to be stable. At the closing stages of the sixth month, the samples stored at 4 °C were found to be more stable than the samples stored at 25 and 45 °C. Table 4 shows the results of the percentage encapsulation efficiency, particle size, and physical appearance of the preparations after 6 months.

**Table 4.** Results of stability studies of unadorned and surface-adorned nanoliposomal formulations.

Time	Initial			1 month			2 months			3 months			6 months		
Parameters	4°C	25°C	45°C	4°C	25°C	45°C	4°C	25°C	45°C	4°C	25°C	45°C	4°C	25°C	45°C
Unadorned gefitinib nanoliposomes															
Encapsulation efficiency, %	87.56±0.17	87.56±0.17	87.56±0.17	78.75±0.56	73.46±1.23	48.27±4.34	70.36±2.98	55.46±2.45	30.02±2.15	61.75±4.67	31.27±3.45	15.36±2.78	61.75±4.67	29.27±3.45	5.36±2.78
Particle size, nm	93.20±3.25	93.20±3.25	93.20±3.25	128.7±3.06	279.5±3.09	528.3±5.45	148.2±4.89	345.5±3.89	8898±4.45	220.8±4.89	1772±10.23	-	420.8±4.89	-	-
Appearance	Clear	Trans-lucent	Milky	*	Milky	*	**			Milky	*	**	-	-	-
Surface adorned gefitinib nanoliposomes															
Encapsulation efficiency, %	88.46±0.97	88.46±0.97	88.46±0.97	83.69±0.65	76.96±2.39	50.79±4.35	81.23±1.64	61.76±2.09	32.461.98	70.56±5.23	32.36±2.34	16.79±3.89	68.56±5.23	30.36±2.34	6.79±3.89
Particle size, nm	132.7±4.56	132.7±4.56	132.7±4.56	139.5±4.65	212.2±4.89	440.8±7.23	153.5±5.67	309.5±4.96	7366±4.12	172.6 ±5.02	1211±12.23	-	372.6±5.02	-	-

*Yellowish suspension with minute bunches of particles discrete on shaking.

**Murky suspension with fungus clusters does not discrete on shaking.

## Discussion

In comparison to other targeted drug delivery systems, nanoliposomes have received the most attention because of their ability to encapsulate a variety of drugs, their low toxicity, good biocompatibility, and lack of immune system activation or suppression [50]. Nanoliposomes may control the rate at which the component they encase is released while shielding the compound from any chemical disturbances in the nearby dispersal medium. Hydrophobic tail and hydrophilic head domains are present in amphiphilic phospholipids. In aqueous media, it created nanoliposomes with a hydrophobic inside and a water-soluble outside.

To optimise parameters including *X*_1_, *X*_2_, and *X*_3_, a total of 17 gefitinib-loaded nanoliposomal formulations were formulated in the current investigation. The centre points, which represent the core values of all components, are also presented. Multiple linear regression analysis was carried out using Design Expert Software (Version 11.0, Stat-Ease, Inc., Minneapolis, MN) to determine the contribution of different parameters for particle size and encapsulation effectiveness. The model was considered significant at *p* < 0.05.

Utilizing Design Expect Software version 11, multiple linear regression analysis was carried out to ascertain how different factors affected particle size and entrapment effectiveness. The data showed that encapsulation efficiency was enhanced at lower *X*_2_, higher *X*_3_ and *X*_1_. The results from counterplots for entrapment efficiency agreed with the Pareto chart's conclusions. The positive value of the estimated coefficients *X*_1_ and *X*_3_ demonstrated the improvement of encapsulation efficiency with the addition of corresponding particulars. The negative values of *X*_2_ demonstrated the augmented entrapment efficiency with the decline in the consequent variable.

All preparations were analysed to estimate their particle dimensions and their particle distribution. In a congealed system, the PDI is generally expressed as a pointer for particle size distribution. The value of PDI recommends a narrow or wide particle size distribution pattern. In the current study, the lower value of PDI demonstrated better consistency and a narrower particle size distribution. The zeta potential values indicated the extent of repellence between similarly charged particles and the stability of vesicular nanoformulations. As the value of zeta potential increased, the charged particles in a dispersal system fended off each other and provided stability against accumulation. Uncharged vesicles can be amassed easily. In the case of nanodispersions, a zeta potential of ±30 is considered optimal for stable formulation. Beyond and beneath this, a caking zone begins that leads to the accumulation of particles [51]. The carboxyl groups on fatty acid moieties and soya lecithins were ionized, leading to a negative charge on particles. Because of the ion-ion interaction between the negatively charged surface of nanoliposomes and the free amino groups of chitosan, the negative charge was transformed into a positive charge [52]. The free amino group of chitosan is responsible for its positive charge in acidic media. The low zeta potential value of the uncoated nanoliposomes may lead to stability problems due to the aggregation of particles. On the other hand, the coated formulation was found to be stable with a high zeta potential value. The results showed that sonication duration had a favourable impact on particle dimension, whereas the cholesterol/soya lecithin and Tween 80/soya lecithin ratios had a negative impact.

To determine the melting point and confirm drug encapsulation in the nanoliposomal formulation before and after coating, thermal analysis of gefitinib, chitosan, cholesterol, soya lecithin, uncoated nanoliposomal, and coated nanoliposomal was carried out using a DSC instrument (Q 10, TA Instruments, New Castle, United States). The lack of an endothermic peak of the drug in the thermogram of formulation indicated that the drug might form an amorphous form, disperse, or liquefy in the polymer medium during the production of nanoliposomes. It confirmed the incorporation of the drug as a molecular dispersal within the nanoliposomes. The endothermic peak in the case of surface-adorned nanoliposomes close to the chitosan liquefaction range confirmed the coating of nanoliposomes by the chitosan.

FT-IR spectroscopy was used to investigate drug excipient compatibility. The frequency and bandwidth of various functional groups were investigated using FT-IR spectroscopy to investigate even minor changes in the structural configurations of the lipid assemblies. The functional moieties on acyl chains and lipid molecules were studied to investigate the arrangement aspect of the lipid molecules in the presence or absence of the chitosan moiety. The results suggested that the drug be stable throughout the encapsulation process [53]. TEM image at a scale bar of 50 nm and a magnification of 200 kV confirmed the coating of chitosan on the surface of nanoliposomes.

Percentage cumulative drug release studies of optimised uncoated (NG13) and surface-adorned nanoliposomal formulations were conducted in three different mediums for 24 h in simulated gastrointestinal fluid (pH 1.2), acetate buffer (pH 4.0), and simulated intestinal fluid (pH 6.8). Slow drug release up to 5 h may be due to a delay in the formation of the hydrated dormant layer by the external shield polymer or to slow drug diffusion from the stagnant layer of the external shell polymer. It is confirmed that the drug is entrapped in the core nanoliposomes. It confirmed the stable incorporation of drugs in formulations, which might help minimize the side effects of drugs and improve the therapeutic index of drugs. It is clear from the results that the release of drug from nanoliposomes was reduced after coating with chitosan at all examined intervals in all three media. Gefitinib release was found to be highest in simulated gastrointestinal fluid because its basic nature makes it more likely to dissolve in an acidic medium. The controlled release of the drug from nanoliposomes in the simulated gastrointestinal fluid may extend its absorption time, which might be beneficial in enhancing the therapeutic index of the drug and reducing its associated side effects.

To recognize the kinetics and mode of drug liberation from nanovesicles, the data obtained from the study of *in vitro* release was integrated into different kinetic equations. A kinetic study of in vitro drug release data demonstrated that the drug acted in accordance with Fickian-type diffusion (*n* < 0.5) in both cases. Based on the value of "*n*", it was found that in both cases, the drug follows the Fickian law of diffusion and diffuses from higher concentrations to lower concentrations. It means that gefitinib was liberated from the nanoliposomes by diffusion mechanism, *i.e*., the solvent penetrated the polymer matrix and caused swelling, ultimately leading to polymer chain disentanglement that relaxed the polymer matrix and liberated the drug by diffusion mechanism. Thus, the polymer chain relaxation and extrication regulated the release of drugs from nanovesicles; it confirmed that both processes, like swelling and solvent penetration, run concurrently at the same rates.

The MTT assay was used to conduct cytostatic studies of gefitinib and uncoated and surface-adorned nanoliposomes on A549 and H1299 cells. MTT assay findings showed that a pure free drug was more effective against cells than an uncoated or chitosan-encrusted formulation. The pure-free drug is more toxic than drug-loaded nanoliposomes in H1299 and A549 cells. Free gefitinib has shown more cytotoxicity than gefitinib-loaded nanoliposomes, demonstrating stable encapsulation of gefitinib by nanoliposomes. The low cytotoxicity of gefitinib-loaded nanoliposomes might be due to the sluggish delivery of the drug from the nanovesicles, which might be beneficial in enhancing the therapeutic index of the drug by avoiding the exposure of a large quantity of the drug. Analogous interpretations were reported by Cheng *et al.* [54]. They reported that the *IC*_50_ value of free cisplatin was less than that of cisplatin-loaded nanoparticles. Zhou et al. demonstrated the stable sequestration of the drug by liposomal formulation, as LGEF-HSPC-2 displayed minimum cytotoxicity compared to the free drug [55]. These findings could help protect cells from all drug exposure and improve the therapeutic index. A higher zeta potential value of coated formulations than unadorned formulations indicates their more anti-leukemic effect. A higher value of positive zeta potential facilitates stronger interactions of nanovesicles with tumour cell membranes, resulting in enhanced cytotoxicity of cancer cells [56]. He *et al.* [57] observed that increased zeta-potential values of carboxymethyl chitosan-attached methyl methacrylate nanoparticles produced a considerable enhancement in the cellular uptake of nanoparticles in murine macrophage cells.

The stability of nanoliposomes was studied at different temperatures as per ICH guidelines to emulate physiological surroundings. During the first month, batches with surface-adorned nanoliposomes did not illustrate any significant variations in particle dimension, entrapment efficiency, or appearance. After 2 months, there was a significant change in particle dimensions, either due to enlargement of the outer layer or adaptation in their morphological characteristics. On the other hand, unadorned nanoliposomes induced significant changes in size immediately after the first month. The coating of chitosan on nanoliposomes was responsible for this assortment in stability among the unadorned and surface-adorned nanoliposomes; its multilayer barrier can obstruct the expansion and discharge of encapsulated content. The stability of plain nanoliposomes has increased after chitosan coating. Thus, by using different concentrations of chitosan solution as coating material, we can develop a suitable sustained drug-release surface-adorned nanoliposomal formulation [58,59].

## Conclusions

Using the Box-Behnken design, the gefitinib-loaded nanoliposomes have been successfully formulated using a reverse-phase evaporation method. It started with the aim of evaluating the effects of three independent variables on the dependent variables of gefitinib-loaded nanoliposomes. In this regard, a Box-Behnken experimental design was used to investigate the consequences of independent variables and to validate the process circumstances during manufacturing. Then, the optimum batch was coated with chitosan. The surface-adorned nanoliposomal formulations have shown a sustained release profile compared to the unadorned optimised nanoliposomal formulation. Reducing the associated side effects could help increase patient compliance and the therapeutic index of the drug. Gefitinib nanoliposomes had shown less cytotoxicity than the free drug against the A549 and H1299 cell lines. This might be due to the sluggish discharge of drugs from nanoliposomes. Because of their higher zeta potential, chitosan-coated nanoliposomes had a slightly higher inhibitory potential than uncoated formulations. Nanoliposomes shielded the encapsulated materials from damage caused by the outside environment. At 4 °C, the content of surface-adorned formulations was found to be more durable than uncoated formulations. It is eminent and acknowledged that the biodistribution of coated nanoliposomes was extensively exaggerated by their surface properties, size, and stability. Therefore, targeting the entrapped gefitinib to particular tissues will be highly favourable. Thus, due to high permeability and increased drug accumulation in cancer cells, chitosan-coated nanoliposomes can be suggested as an appropriate nanocarrier to regulate the cancer cells' expansion.
